# Supplementary material for: Optical flow estimation from event-based cameras and spiking neural networks
Source: Front Neurosci. 2023 May 11;17:1160034. doi: 10.3389/fnins.2023.1160034 (PMC10210135; doi:10.3389/fnins.2023.1160034)
Supplement: Supplementary file 1 [file Data_Sheet_1.PDF]

Supplementary Material

1 SUPPLEMENTARY DATA

1.1 Upsampling techniques illustration

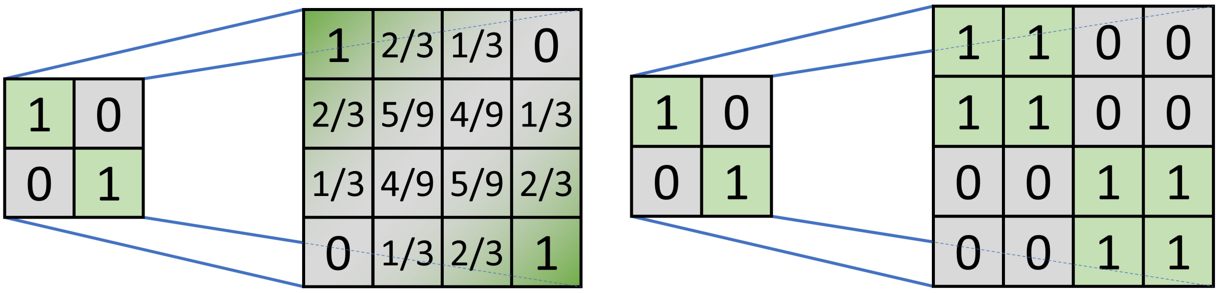

Figure 1a. Upsampled tensor via Bilinear Upsampling.

Figure 1b. Upsampled tensor via Nearest Neighbor Upsampling.

Figure 1. Upsampling techniques comparison. Unlike Bilinear Upsampling, Nearest Neighbor Upsampling guarantees binary tensors after the upsampling operation, being therefore implementable on neuromorphic hardware.

1.2 Train and validation split

Here is the sequence division we have done for our train and validation split:

- Trainin split:
  - zurich_city_01_a
  - zurich_city_02_a
  - zurich_city_02_c
  - zurich_city_01_e
  - zurich_city_05_a
  - zurich_city_05_b
  - zurich_city_06_a
  - zurich_city_07_a
  - zurich_city_09_a
  - zurich_city_10_a
  - zurich_city_10_b
  - zurich_city_11_a
  - zurich_city_11_c
- Validation split:
  - thun_00_a
  - zurich_city_02_d
  - zurich_city_03_a
  - zurich_city_08_a
  - zurich_city_11_b

The data split has been performed in order to ensure around 75% of the available data beeing used during training, while the remaining 25% was used in evaluation.

1.3 Result Plots

Here are the plots containing the results of our trainings, evaluated on our validation split. Each plot consists of the modification of a single parameter or architectural block, for better comparison of their effect on performance.

2 SUPPLEMENTARY TABLES AND FIGURES

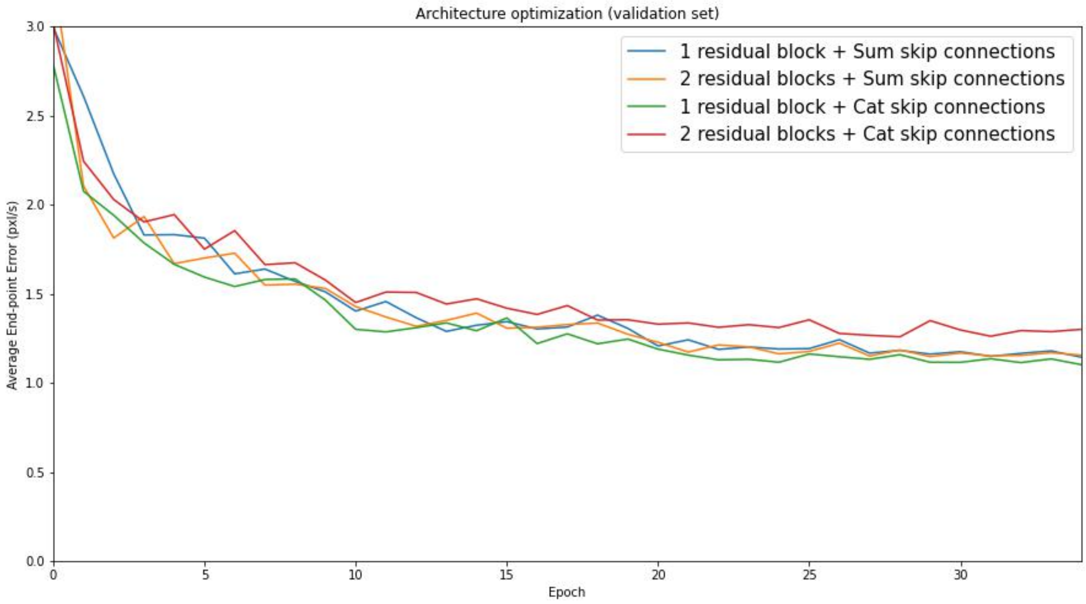

Figure S2. Architecture optimization: 1 vs. 2 residual blocks in the bottleneck,, and Sum vs. Cat skip connections. We have found the best architecture to consist of CAT skip connections and a single residual block, which amount to a total of 1.2 million parameters for our base model.

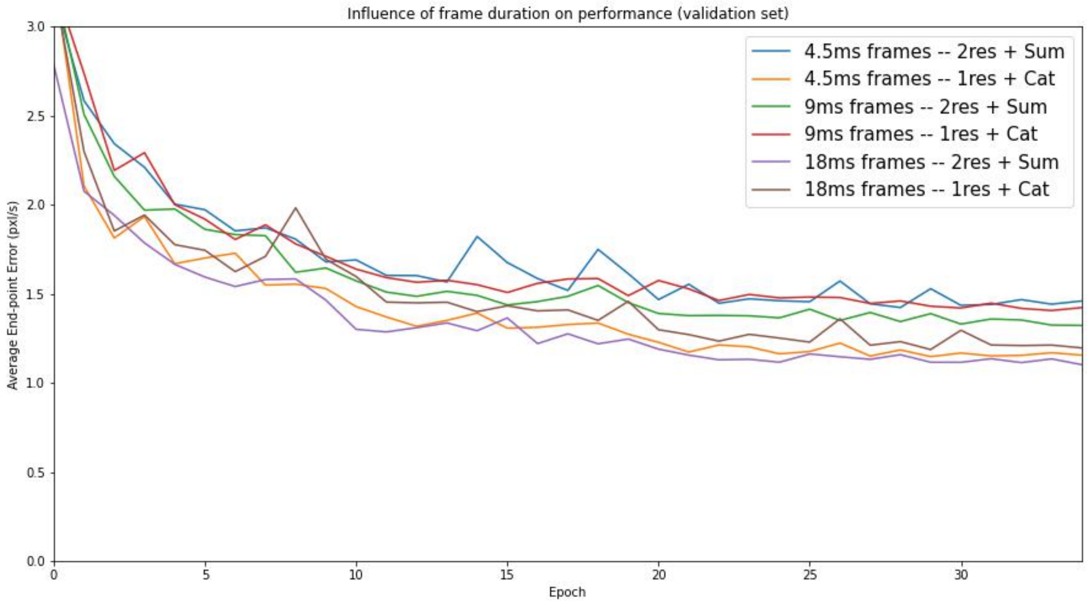

Figure S3. Frame duration optimization: 4.5ms, 9ms and 18 ms. We find intermediate frames to be the optimal choice when it comes to accuracy, since shorter histograms do not manage to capture sufficiently long-term time features, and longer histograms are not sufficiently crisp.

2.0.1 Ablation Studies

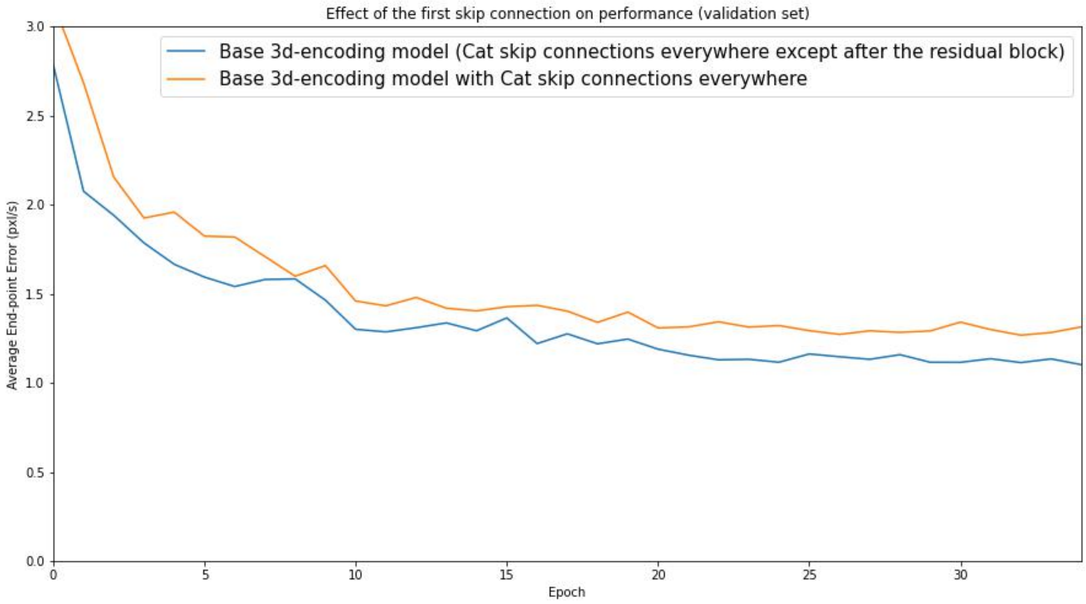

Figure S4. Accuracy comparison between strided convolutions and strided maximum pooling as downsamplnig strategies. We show that our approach, where individual spikes carry less importance (one single spike within the kernel region is enough to forward the information) achieves remarkably better results.

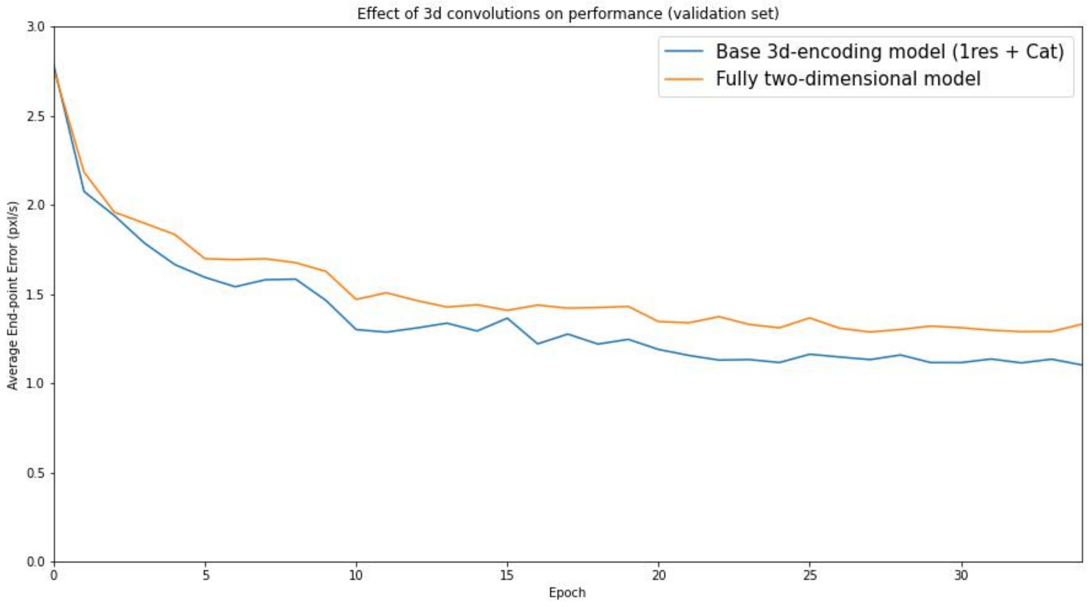

Figure S5. Accuracy comparison between our proposed 3d-encoder architecture and an equivalent fully 2-dimensional architecture. We show that explicitly handling the temporal dimension with consecutive convolutions along a temporal axis yields better quality optical flow estimations.

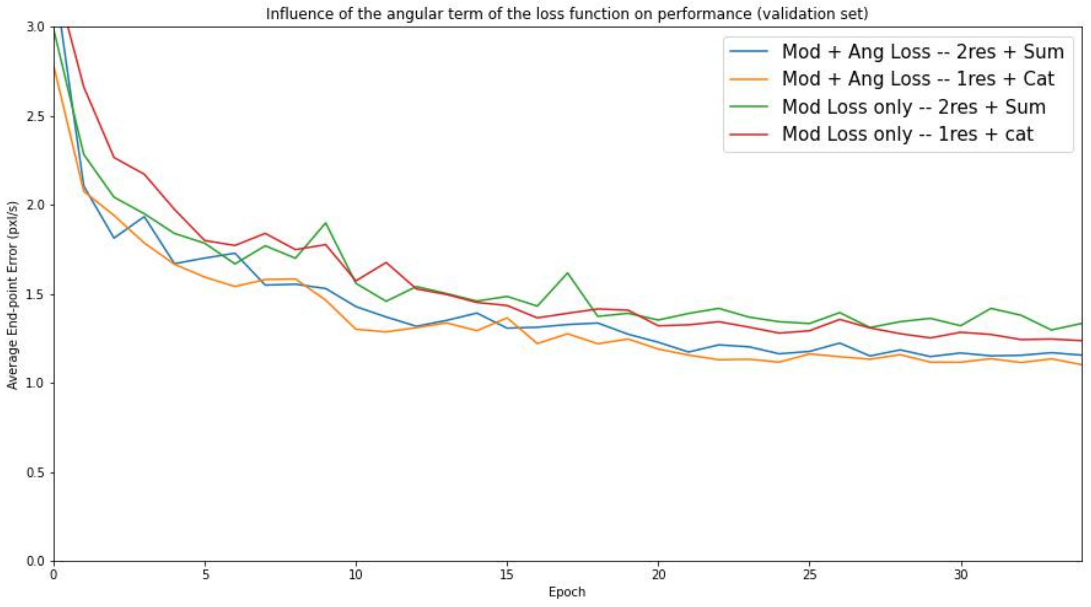

Figure S6. Influence of the angular term in the loss function on the achieved accuracy. We can observe that enforcing this term on the loss evaluation helps the neuron acquire a better general understanding of the scene, and therefore achieve better quality estimations.

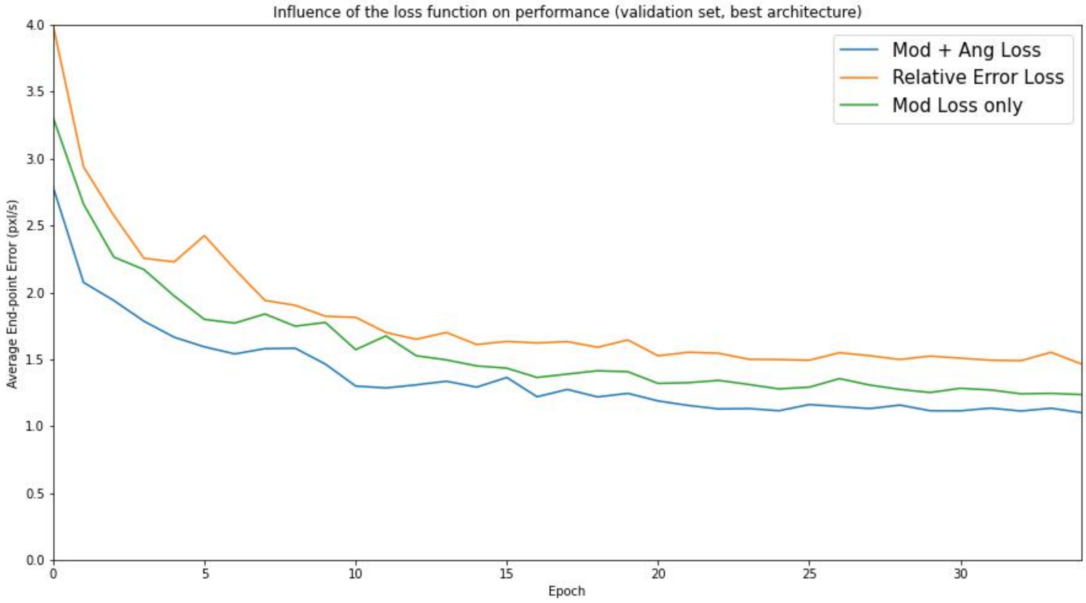

Figure S7. Influence of the loss function on the achieved accuracy (validation set): best loss model (Mod + Ang), norm of the error vector (only error norm) and relative error (error norm divided by the optical flow ground-truth magnitude).

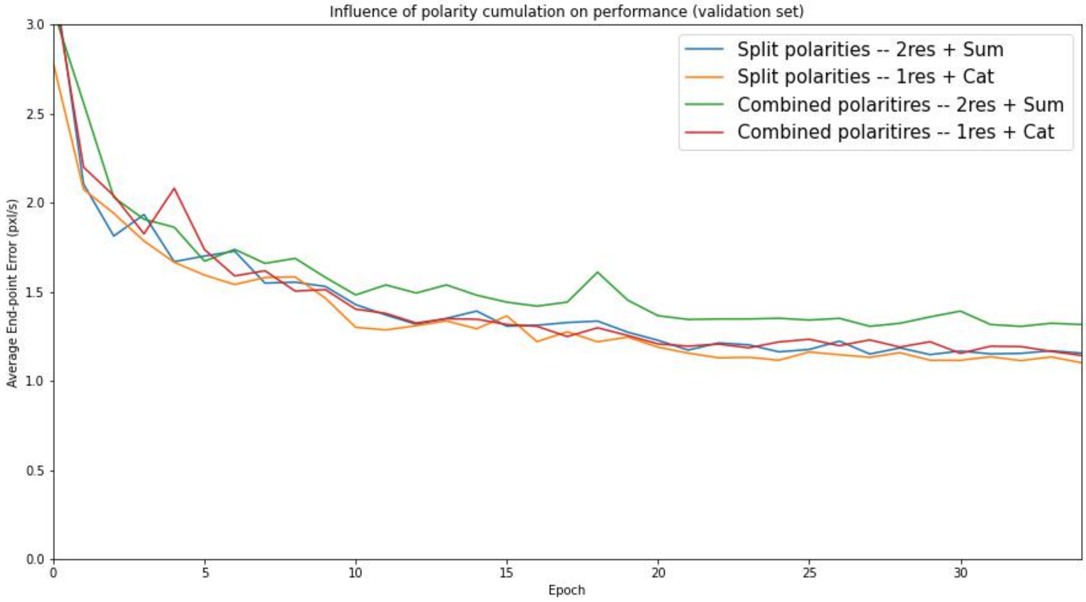

Figure S8. Effect of combining the polarities in a single channel on the final validation performance. We can observe that keeping split polarities yields better performance, although not very significantly in the case of our best architecture.

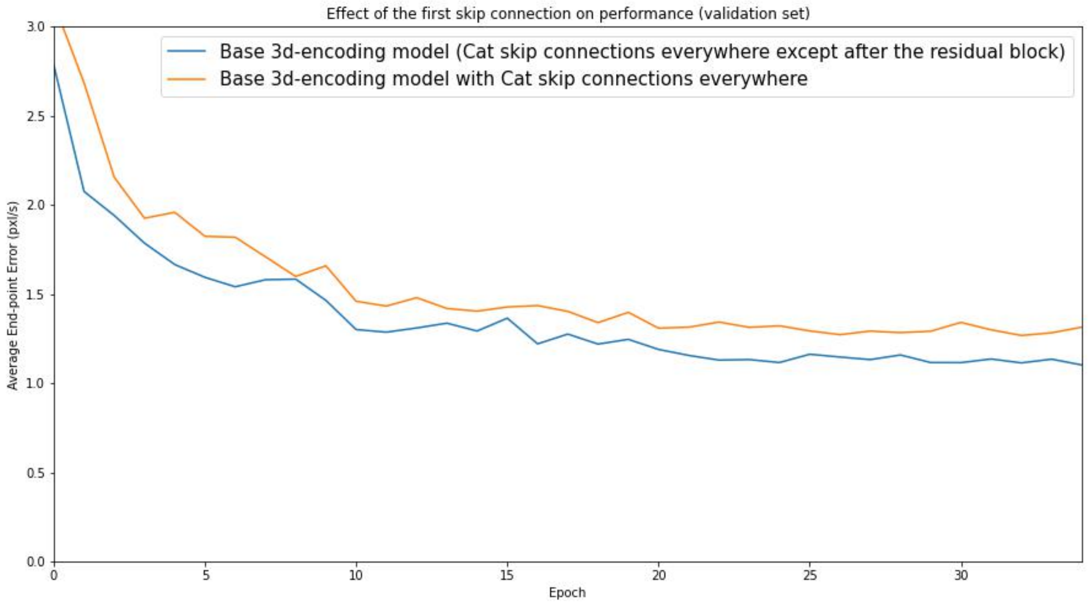

Figure S9. Influence of the first skip connection (last encoder with fisrt decoder) on the model's performance: the blue curve represents our base model, whereas the yellow curve represents a CAT skip connection between linking the encoder's output and the decoder's input.
